# Impact of HbA1c Reduction on Major Kidney Outcomes in Type 2 Diabetes With Poor Glycemic Control and Advanced CKD

**DOI:** 10.1155/ije/9919963

**Published:** 2025-05-04

**Authors:** G. Navarro-Blackaller, A. S. Benitez-Renteria, K. Hernández-Morales, J. Rico-Fontalvo, R. Daza-Arnedo, G. G. Gómez-Ramírez, J. R. Camacho-Guerrero, M. A. Pérez-Venegas, J. Carmona-Morales, A. N. Oseguera-González, C. Murguía-Soto, G. Chávez-Alonso, F. García-Peña, C. J. Barrera-Torres, E. Orozco-Chan, M. Arredondo-Dubois, A. Martínez Gallardo-González, J. A. Gómez-Fregoso, F. G. Rodríguez-García, V. H. Luquin-Arellano, G. Abundis-Mora, L. Alcantar-Vallin, R. Medina-González, G. García-García, J. S. Chávez-Iñiguez

**Affiliations:** ^1^Nephrology Service, Hospital Civil de Guadalajara Fray Antonio Alcalde, Guadalajara, Jalisco, Mexico; ^2^University of Guadalajara Health Sciences Center, Guadalajara, Jalisco, Mexico; ^3^AstraZeneca Mexico, Medical Affairs, Mexico City, Mexico; ^4^Kidney, Diabetes, and Metabolism Committee, Colombian Association of Nephrology and Hypertension, Bogotá, Colombia; ^5^Departamento de Nefrología, Facultad de Medicina de la Universidad Simón Bolívar, Barranquilla, Colombia

**Keywords:** chronic kidney disease, diabetes, kidney replacement therapy, major adverse kidney events, mortality

## Abstract

**Aims:** In subjects with type 2 diabetes (DM), poor glycemic control, and advanced chronic kidney disease (CKD), the kidney benefit of the reduction of glycated hemoglobin (HbA1c) is not well established.

**Methods:** In a retrospective cohort, we included patients with DM, CKD grade 3b-5, and HbA1c > 9% to evaluate the risk of developing major adverse kidney events (MAKE) defined as the start of kidney replacement therapy (KRT), ≥ 25% or ≥ 40% decline in the glomerular filtration rate (eGFR) from baseline, and death; patients were divided according to the HbA1c levels at the end of the follow-up into the following groups: > 75 mmol/mol (≥ 9.0%), 74–64 mmol/mol (8.9%–8.0%), 64–53 mmol/mol (7.9%–7.0%), and < 52 mmol/mol (< 7.0%). We described their characteristics and analyzed their risks, adjusting for confounding variables.

**Results:** From 2015 to 2023, 111 patients were included. In 46 patients (41.4%), the HbA1c at the end of follow-up (60 months) was still > 75 mmol/mol (≥ 9%), and each patient had a mean of 4.9 HbA1c measurements. The mean age was 59 years, and 46% were male; the baseline eGFR was 25 mL/min/1.73 m^2^. MAKE occurred in 67% of cases. In a multivariate analysis, the risk of MAKE was not associated with the HbA1c groups, nor was it associated with any of the MAKE components individually, nor in certain subgroups. When evaluating the magnitude of percentage changes in HbA1 with the initiation of KRT, we did not find any association.

**Conclusions:** With advanced CKD and poor glycemic control, changes in HbA1c during long follow-up are not associated with MAKE or its individual components.

## 1. Background

Diabetes is the most common cause of chronic kidney disease (CKD) in the world, in addition to its well-known association with an increased cardiovascular (CV) risk [1–3]. Within the multiple pathophysiological mechanisms that promote the deterioration of tissue function, glycemic control has historically been one of the most studied [4], specifically due to its association with a decrease in the glomerular filtration rate (GFR), the appearance of albuminuria [5], and increased CV comorbidity [6]. Hyperglycemia involves different metabolic pathways that culminate in intense inflammation, complement activation, and fibrosis, which severely deteriorate kidney tissue [5, 7–10]. Measurements of glucose in peripheral or central blood have been used to evaluate the control of diabetes, but the metric used for chronic (> 3 months) monitoring of glucose values is glycosylated hemoglobin (HbA1c), which, despite its limitations, is still recommended by diabetes and CKD management guidelines [11]. According to one study, HbA1c values > 75 mmol/mol (≥ 9.0%) are considered to indicate poor glycemic control and have been associated with practically all negative kidney outcomes [12]; on the other hand, a decrease in HbA1c during follow-up improves kidney prognosis [13]. These findings are not consistent when studying people with advanced CKD, where HbA1c values considered to be outside of the normal range do not have the same negative association compared to what is observed in people with healthy kidneys [14, 15]; this information has allowed for more laxity in glycemic control [16, 17]. Information regarding the association between HbA1c values and cardiorenal events in people with good or moderate kidney function has been published [18]. However, that study excluded the most vulnerable patients with worse kidney function; therefore, evidence showing that a decrease in HbA1c in people with poorly controlled diabetes and advanced CKD is associated with a reduction in major adverse kidney events (MAKE) is scarce [16]. The effects of HbA1c reductions to optimize clinical outcomes in these patients are unknown and represent an important research topic [19]. We hypothesized that the potential detrimental consequences of strict glycemic control may be associated with increased MAKE among advanced CKD patients.

To contribute to this research area and narrow the associated information gap, we studied a cohort of patients with advanced CKD and poor glycemic control who, during a long follow-up period, achieved different HbA1c values, and we sought to determine whether those trajectories were associated with MAKE.

## 2. Material and Methods

This is a retrospective cohort study conducted at the Hospital Civil de Guadalajara Fray Antonio Alcalde, Mexico, between April 2015 and February 2022, a university academic center with 1000 beds. All patients considered were under the care of the nephrology service and attended the Renal Health Clinic, an interdisciplinary program that involves a nephrology nurse, a nutritionist, a psychologist, and a nephrologist. The Renal Health Clinic has the purpose of preventing the progression of CKD and avoid CV events, optimizing the management of comorbidities and educating patients about kidney replacement therapy (KRT) modalities, from a multiparametric and comprehensive perspective. To this study, we only focused on patients with CKD G 3b, 4, and 5 without dialysis, with diabetes and HbA1c > 9% at baseline. The inclusion criteria were the presence of the CKD stage 3b, four, or 5, being > 18 years old, having a diagnosis of type-2 diabetes mellitus, having HbA1c values > 75 mmol/mol (> 9%), and having at least 3 HbA1c measurements recorded during follow-up. Patients with KRT, pregnancy, attributable causes of CKD other than diabetes, and those without follow-up examinations were excluded. CKD was defined according to the KDIGO guidelines using the glomerular filtration rate (eGFR) estimated by the CKD-EPI equation of less than 60 mL/min/1.73 m^2^ or any marker of kidney disease for more than 3 months. CKD G3b, G4, and G5 were defined as an eGFR of 44–30 and 29–16 mL/min/1.73 m^2^ and < 15 mL/min/1.73 m^2^, respectively [20].

Our primary objective was to analyze whether there was an association between the change in HbA1c to values considered indicative of better control during a specified time-frame and the composite outcome of MAKE, defined as the start of KRT, worsening of kidney function denoted by ≥ 25% (MAKE^∗^) or ≥ 40% (MAKE^∗∗^) decline in the eGFR from baseline, and death. We analyzed the individual components of MAKE as a secondary objective. Additionally, as exploratory analyses, we considered whether there were subgroups of patients who achieved some benefits from reaching specific HbA1c values, the percentage change in the value of HbA1c, and its association with these events. The subgroups considered were the CKD stage (G3b, G4, and G5), the hypertension status, and the body mass index (BMI: underweight, normal weight, overweight, and obesity). KRT was defined as the initiation of peritoneal dialysis or hemodialysis. All-cause mortality was defined by the date of death from linked death certification records. No financial compensation was provided.

This study was funded by a grant from the Secretaria de Salud Jalisco y el Consejo Nacional de Ciencia, Humanidades y Tecnología CONAHCYT.

Written informed consent was obtained from participants, and this consent grants permission to use personal data for clinical research purposes. The study was approved by the Hospital Civil de Guadalajara Fray Antonio Alcalde Institutional Review Board (HCG/CEI-0550/15). This study was reported according to the Strengthening the Reporting of Observational Studies in Epidemiology (STROBE) reporting guidelines [21] and the REporting of studies Conducted using Observational Routinely collected health Data (RECORD) statement [22].

### 2.1. Data Collection

Clinical characteristics, demographic information, and laboratory data were collected using automated retrieval from the institutional electronic medical record system. MAKE was the primary outcome. Demographic and clinical variables were collected, including age, the duration of diabetes, blood pressure, hypothyroidism, CKD stage, eGFR, smoking, cerebrovascular disease, ischemic heart disease, and prespecified biochemical data, such as HbA1c, hemoglobin, cholesterol, serum albumin, proteinuria, platelets, leukocytes, glucose, urea, creatinine, sodium, potassium, chloride, phosphate, and calcium. Antihypertensive and antidiabetic therapies were commonly prescribed. Categorization of HbA1c levels: Participants were categorized into four groups based on HbA1c values: > 75 mmol/mol (≥ 9.0%), 74–64 mmol/mol (8.9%–8.0%), 64–53 mmol/mol (7.9%–7.0%), and < 52 mmol/mol (< 7.0%). Indications for starting KRT were fluid overload resistant to diuretics, severe hyperkalemia, severe metabolic acidosis, and uremic manifestations, including encephalopathy, pericarditis, and seizures [23–25].

### 2.2. Statistical Analysis

The statistical analysis for baseline characteristics was conducted to assess the relationships between different categories of HbA1c levels and various clinical, demographic, and laboratory parameters. Descriptive statistics were performed using means and standard deviations (SD) for continuous variables, while frequencies and percentages were reported for categorical variables. Comparative analysis was developed to compare the differences across the HbA1c categories. One-way ANOVA was used for continuous variables, and chi-squared tests were employed for categorical variables. Where appropriate, trend analysis was performed to evaluate the linear relationship between ordered groups, and the *p* values for the trend were reported. We opted for a *Z* test for comparison of proportions in this case, given that our data represent two independent groups (those with HbA1c changes less than zero and those with changes greater than/equal to zero). The *Z* test is appropriate here because it is designed to test hypotheses about the difference in proportions between two independent groups, particularly when sample sizes are large enough to meet the test's assumption of normality for binomially distributed data.

To retrospectively justify the sample size, we employed the standard cohort study sample size formula. We assumed a significance level (α) of 0.05 and a statistical power of 80%. The actual sample size of 111 patients in the study is sufficient for detecting differences in comparing the HbA1c > 75 mmol/mol (≥ 9.0%) group, versus the other final subgroups (sample size of 74, 51, and 74 for < 52 mmol/mol (< 7.0%), 64–53 mmol/mol (7.0%–7.9%), and 74–64 mmol/mol (8.0%–8.9%), respectively).

A *p* value of less than 0.05 was considered to indicate statistical significance. All tests were two-sided. These methods ensured a comprehensive assessment of the data, enabling the identification of significant trends and differences across the HbgA1c categories about the studied variables in baseline characteristics.

The statistical analysis aimed to assess the odds ratios (OR) of primary and secondary objectives in relation to various HbA1c levels during follow-up. Calculation of OR: For each category, the OR were computed with 95% confidence intervals (CIs) for primary and secondary objectives. Statistical tests: Logistic regression models were employed to calculate the OR, adjusting for potential confounding factors as needed. The reference category for the analysis was the HbA1c > 75 mmol/mol (≥ 9.0%) group, as indicated by an OR of 1.0. A *p* value of less than 0.05 was considered indicative of statistical significance. A polynomial regression model was employed to capture the nonlinear relationship between the eGFR and the visit number across different final HbA1c groups. This analytical approach provided a robust way to represent the complex dynamics of eGFR changes during follow-up.

A second-degree polynomial was chosen for the regression model, allowing for a parabolic relationship between the independent variable (visit number) and the dependent variable (eGFR). This choice added flexibility to the model by incorporating both linear and quadratic terms, reflecting the possibility of curved patterns in the data. The model was fitted separately for each HbA1c group using the following general form of a second-degree polynomial equation:

The model was fitted separately for each HbA1c group using the following general form of a second-degree polynomial equation:

(1) $$\begin{array}{c} \text{eGFR}=\beta 0+\beta 1\cdot \left(\text{Visit Number}\right)+\beta 2\cdot \left({\text{Visit⁣Number}}^{2}\;\right). \end{array}$$

*β*0, *β*1, and *β*2 are the coefficients estimated from the data, and the squared term allows for capturing curvature in the relationship. The resulting polynomial regression lines were plotted against the visit numbers to visualize the eGFR trends for each HbA1c category.

In the subgroup analysis by final HbA1c, forest plots were used to display the OR in a logarithmic scale, with horizontal lines indicating the 95% CIs and individual points marking the exact ORs. The plots were organized by the event and provided a comprehensive visual representation of the relationships between the events by each subgroup.

We assessed the time between the first measurement of HbA1c and KRT, grouped by different categories of HbA1c values. First, missing values in the time interval between the first HbA1c measurement and KRT were addressed. Any missing values were replaced with the maximum follow-up time observed in the dataset, and the corresponding indicator was set to zero, reflecting the absence of the event. Kaplan–Meier survival curves were then plotted for each HbA1c category. The Kaplan–Meier estimator is a nonparametric statistic used to estimate the survival function from lifetime data, which is the time until the initiation of KRT.

Patients were categorized into four groups based on their HbA1c percentage change from baseline to the last visit, and ORs with 95% CIs were computed to measure the risk of starting KRT during the follow-up period. The category of “−1.5–0” change in HbA1c was used as the reference category, with an OR set to 1.0.

The data were categorized into distinct groups based on the percentage change in HbA1c. For each group, the OR with a corresponding 95% CI was calculated, reflecting the odds of starting KRT relative to the reference group. We utilized Python 3.11 and RStudio 4.3 for statistical analysis.

## 3. Results

**Figure 1:**
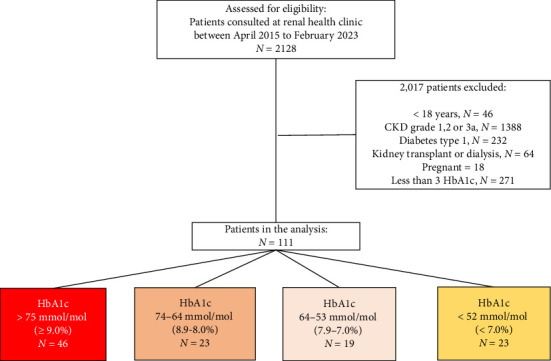
Flowchart of the cohort.

Figure 1 shows the study flowchart of the entire cohort. During the period from April 2015 to February 2023, a total of 2128 patients attended the Renal Health Clinic, and 2017 were excluded for not meeting the inclusion criteria, leaving a total of 111 patients. They were stratified for the final analysis according to the HbA1c value reached at the last visit, and 46 (41.4%), 23 (20.7%), 19 (17%), and 23 (10.7%) patients reached HbA1c values of > 75 mmol/mol (≥ 9.0%), 74-64 mmol/mol (8.9%–8.0%), 64–53 mmol/mol (7.9%–7.0%), and < 52 mmol/mol (< 7.0%) at the end of the follow-up, respectively. The demographic and clinical characteristics of the total cohort are shown in Table 1. The mean baseline HbA1c was 92 mmol/mol (10.6%), and the final HbA1c of the total cohort was 71 mmol/mol (8.7%), with an average of 4.9 (SD 3.0) measurements per patient during 12 visits. Interestingly, less than half of the patients (41%) maintained an HbA1c > 75 mmol/mol (≥ 9.0%) during follow-up. The remaining patient characteristics were as follows: the mean age was 59 [13] years old; 46% were male; the mean eGFR was 25 mL/min/1.73 m^2^; more than half had CKD stage 4 (54%). The proportions of patients who achieved HbA1c values > 9.0%, 8.9%–8.0%, 7.9%–7.0%, and < 7.0% during follow-up according to the CKD stage were G3b 34%, 34%, 26%, and 43%, respectively; G4 56%, 56%, 57%, and 52%, respectively; and G5 8.7%, 8.7%, 15.8%, and 4.3%, respectively. The mean proteinuria per day was 4.28 g; most patients had anemia, with a hemoglobin level of 10.59 g/dL; the vast majority used insulin (85%); and only 19% used SGLT2 inhibitors. The frequency of KRT initiation during follow-up was 27.9%.

As expected, demographic and clinical characteristics differed between groups stratified by the HbA1c levels reached over the 60 months of follow-up, as shown in Table 1. Among the most relevant, we found that those who reached lower HbA1c values < 52 mmol/mol (< 7.0%) were more overweight and obese. Comorbidities such as stroke, acute myocardial infarction, cancer, and hypothyroidism were similar among the HbA1c groups, as were hemoglobin, glucose, urea, creatinine, sodium, chloride, phosphate, albumin, cholesterol, and triglycerides. More patients in CKD G3b reached lower HbA1c values, and the opposite effect was observed in CKD G4. The severity of proteinuria was more commonly found in those with HbaA1c reaching lower values. Regarding treatment, more patients who achieved lower HbA1c levels consumed more renin angiotensin aldosterone system inhibitors, β-blockers, calcium antagonists, and statins. It is no wonder that the frequency of insulin use was more prevalent in those with higher HbA1c at baseline, and iSGLT2 was less often prescribed in those with lower HbA1c values. About one-third of patients had an episode of hypoglycemia, which was more frequent in the groups with lower HbA1c levels (*p* trend = 0.026) (Table 1).

### 3.1. Relationships Between HbA1c Groups and Study Outcomes

In the total cohort, at the end of follow-up, the eGFR decreased to 10 mL/min/1.73 m^2^, and the eGFR trajectory decreased similarly between the HbA1c groups, with no difference between them (*p* ≥ 0.05 for all) (Supporting Figures 1A and 1B).

MAKE occurred in 74 (67%) of patients. The frequency of MAKE^∗^ when analyzed according to the different HbA1c groups was 28 (60%), 16 (69%), 14 (73%), and 16 (69%) in > 75 mmol/mol (≥ 9.0%), 74–64 mmol/mol (8.9%–8.0%), 64–53 mmol/mol (7.9%–7.0%), and < 52 mmol/mol (< 7.0%), respectively (Supporting Table 1 and Supporting Figure 2).

**Figure 2:**
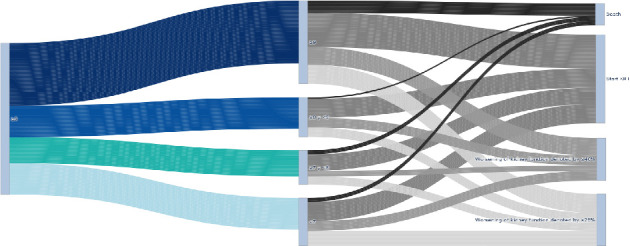
Alluvial diagram shows the frequencies of each of the individual MAKE component according to changes in HbA1c categories at the end of follow-up.

The primary objective, which was to determine the association between changes in HbA1c and the risk of MAKE, is presented in Table 2. We found that the risk of MAKE was not associated with changes in HbA1c at the end of the follow-up (*p* > 0.05 for all). The alluvial diagram in Figure 2 shows how the cohort transitioned according to HbA1c values at the end of follow-up and how they were distributed in the individual components of the MAKE composite.

**Figure 3:**
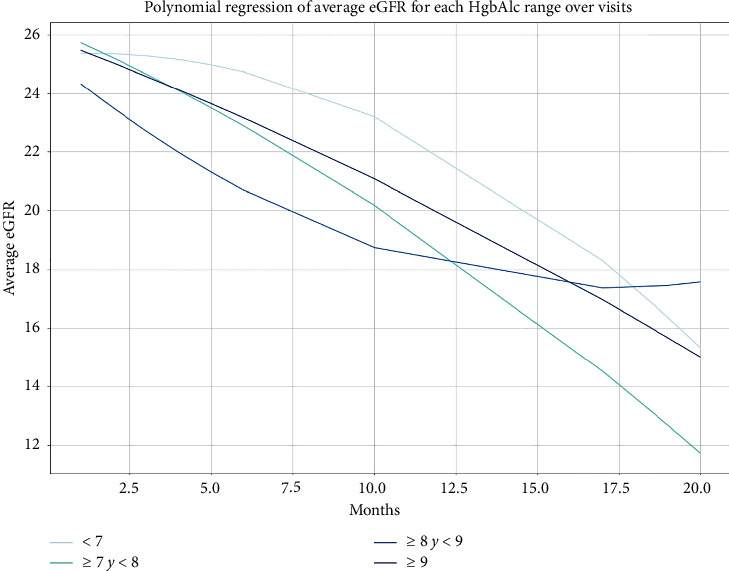
Polynominal regression of the average eGFR for each HbA1c group over months.

In the analyses of the secondary objectives, no associations were found between the risks of each of the individual MAKE components and changes in HbA1c, as shown in Table 2 and Figure 3. Mortality analyzed as the accumulated survival during the 60 months of follow-up was similar among the HbA1c groups (Supporting Figure 3).

We found that the magnitude of the percentage change in HbA1c, taking as a reference the −1.5%–0% group, has no association with an increased risk of KRT (*p* for all) (Table 3).

We performed a subgroup analysis to identify whether there were certain patients who presented an increased risk of the MAKE composite or any of its components. The subgroups considered were CKD by grades (G3b, G4, and G5), hypertension status, and the BMI (underweight, normal weight, overweight, and obesity). We observed that none of those categories had a significant association with the MAKE composite or any of its individual components.

## 4. Discussion

In our retrospective cohort of patients with advanced CKD, diabetes, and poor glycemic control, we observed that the change in HbA1c during long-term follow-up was not associated with the risk of composite MAKE or any of its individual components.

We found that reducing the HbA1c value of a patient considered to be in poor glycemic control > 75 mmol/mol (≥ 9.0%) was not associated with a change in the risk of MAKE. The effect of reducing HbA1c in people with diabetes and advanced CKD on kidney evolution is unknown and has not yet been answered satisfactorily [19]. Glucose and insulin metabolism in patients with diabetes are profoundly altered by advanced CKD. Patients with advanced CKD have been systematically excluded from the largest clinical trials that have studied the association between strict glycemic control and kidney events, significantly limiting the understanding of this potential benefit [26–28].

Our findings on the absence of an association between the risk of MAKE and changes in HbA1c are comparable to the results of the DNETT-Japan clinical trial, in which patients with CKD G3b were randomized to strict control of HbA1c for 5 years. Compared with those in the nonstrict control group, no benefit was observed for the MAKE composite [29], which is in line with what was found among the participants in our cohort. Notably, our study has different characteristics from those in the DNETT-Japan study, and we included patients with worse kidney function and higher HbA1c values (the mean eGFR 25 mL/min/1.72 m^2^ and HbA1c 10.6%). In addition, our patients had a greater prevalence of CV comorbidities. Despite these important differences, the results for MAKE were very similar.

When analyzing the MAKE components individually, we also found no associations of those components with changes in HbA1c, a result similar to that found by Shakita et al. [29]. The absence of an association between the HbA1c value and kidney events has also been observed in other cohorts of patients with better kidney function [14, 15]. These studies differ from ours in the baseline HbA1c value. In our study, all patients had poor glycemic control > 75 mmol/mol (≥ 9.0%), at baseline, which makes our cohort a relevant novelty, unlike previous studies where HbA1c was much smaller; therefore, it is impossible to make a fair comparison.

It is noteworthy that although all our patients had poorly controlled glycemic control (HbA1c > 75 mmol/mol, ≥ 9.0%), 41% of them maintained the same values during follow-up. This event could be explained by the influence of studies that have disseminated information on the high frequency of hypoglycemia in this highly vulnerable group of patients [30, 31], which could have influenced nephrologists to raise some concern regarding the intensification of glycemic control. Our patients were comorbid with poor kidney function and CV complications. This patient phenotype is more fragile and susceptible to hypoglycemia [13]. It is noteworthy that in our cohort, one in three patients experienced some hypoglycemic events during follow-up, which occurred more frequently in the groups where HbA1c was reduced the most. These events are consistent with those described in the literature [13, 30, 31]. The risk of hypoglycemia in advanced CKD is exacerbated by the failure of gluconeogenesis in the failed kidney as well as reduced clearance of many antihyperglycemic agents, particularly insulin. Our study has clinical implications, and the evidence for current guideline recommendations derives from clinical trials that focus on HbA1c as the definitive measure of the efficacy of an intervention. For instance, intensive glycemic control to achieve near normoglycemia delayed the onset and progression of albuminuria in patients with diabetes and normal kidney function, supporting the conclusion that glycemic control itself helps prevent CKD and its progression. This premise has not been proven to occur in people with advanced CKD, and our findings support the KDIGO guidelines for the management of diabetes in these specific populations, which emphasize that comorbid and vulnerable patients could be laxer with glycemic control [16], thus avoiding hypoglycemia and polypharmacy and improving quality of life.

We found a nonsignificant trend between HgA1c percentage changes and KRT start. This findings included those patients who at the end of the study had HbA1c values < 48 mmol/mol (< 6.5%), which would mean that the subjects were exposed to periods of hypoglycemia [30, 31] and had an increased risk of negative events that can impair kidney function, such as sympathetic hyperactivity, increased counterregulatory hormones, and sustained inflammation [32, 33]. This finding is consistent with what was previously reported by Jung, where they identified that in patients with CKD G3-4 and diabetes, fasting glycemia levels less than 120 mg/dL (< 48 mmol/mol, < 6.5%), were associated with a significantly increased risk of a composite outcome, including the doubling of serum creatinine, end-stage kidney disease (ESKD), or death from CKD [12]. On the other hand, Navaneethan et al., in a cohort of patients with diabetes and CKD G1-5, a 2.3 year follow-up and competing-risk models, revealed that the baseline HbA1c value was not associated with ESKD [14]; this study differs from ours in which we focused on the final values of HbA1c, not on the trajectory.

It is possible that improving HbA1c during advanced CKD could offer benefits for other complications of diabetes, such as CV events, retinopathy, or neuropathy. These objectives are beyond the scope of our study and therefore are not discussed.

The strengths of our study include our cohort, which included patients with advanced CKD and poor baseline glycemic control, and our long follow-up period (60 months), which makes this study unique. These strengths must be contrasted with the limitations of the study. The nature of our retrospective study design means that our cohort could only be analyzed for association, not for causality. It was a small cohort, but the patients were predominantly CKD G4 and had HbA1c > 75 mmol/mol (≥ 9%), a combination that is not frequently found in daily clinical practice due to the effects of worsening kidney function on glucose metabolism. Some of the subgroups contained a very small number of patients, which limited the analysis and interpretation of our results. There were no patients with the GLP-1 receptor antagonist; with this treatment, the effect on MAKE could have been different. The KDIGO guideline for the management of diabetes and CKD comment that with elevated HbA1c, insulin, or GLP-1 receptor antagonist can be started [16]. Having considered HbA1c as a measure of glycemic control, these tests may be less reliable in persons with advanced CKD owing to the influence of CKD-related anemia and the lifespans of red cells with subsequent alterations in hemoglobin glycation percentages [34]. We did not use other glycemic control markers such as fructosamine and glycated albumin. It is possible that those markers would have been more useful in accurately evaluating glycemic control in our population since it is known that high urea levels and metabolic acidosis could increase HbA1c [35]. In our center, we do not have access to continuous glucose monitoring devices that are more precise in glucose control and predict its complications; however, there are a limited number of studies that used those devices in the CKD population [36]. Finally, only episodes of hypoglycemia reported in the central laboratory results on the days of the visit were described; since episodes of hypoglycemia that occurred at home with ambulatory monitoring were not reported, the reporting of this complication may have been under-evaluated, and this is relevant because hypoglycemia is associated with a stricter control of HbA1c [30, 31]. This work was presented as a poster at the ASN 2024 conference [37].

## 5. Conclusions

In summary, in people with advanced CKD and poor glycemic control, changes in HbA1c during a long follow-up period are not associated with MAKE. Our study generated hypotheses and can serve as a reference for conducting a clinical trial to investigate the association between the decrease in HbA1c and MAKE in this highly vulnerable population.

## Figures and Tables

**Table 1:** Baseline clinical characteristics of patients with CKD, diabetes, and HbA1c > 9% stratified according to final HbA1c values.

Variable	Total	HbA1c > 75 mmol/mol (≥ 9.0%)	HbA1c 74–64 mmol/mol (8.9%–8.0%),	HbA1c 64–53 mmol/mol (7.9%–7.0%)	HbA1c < 52 mmol/mol (< 7.0%)	*p* value, trend
Total (*N* [%])	111 (100)	46 (41.4)	23 (20.7)	19 (17.1)	23 (20.7)	0.004, 8.204
Age (years), mean (SD)	59.13 (13.86)	59.20 (14.3)	57.57 (14.6)	60.21 (11.1)	59.65 (14.8)	1.000, 8.204
Male (*N* [%])	52, (46.8)	21, (45.6)	9, (39.1)	10, 52.6	12, 52.1	0.930, 1.465
Underweight (*N* [%])	3 (6.52)	3 (6.52)	0 (0)	0 (0)	0 (0)	1.0
Normal weight (*N* [%])	28 (25.23)	14 (30.4)	5 (21.74)	6 (31.58)	3 (13.04)	0.018, 0.025
Overweight (*N* [%])	51 (45.95)	18 (39.1)	10 (43.48)	9 (47.37)	14 (60.87)	0.001, 0.011
Obesity (*N* [%])	29 (26.13)	11 (23.9)	8 (34.7)	4 (21.05)	6 (26.09)	0.001, 0.026
Hypertension (*N* [%])	99 (89.19)	44 (95.6)	18 (78.2)	17 (89.47)	20 (89.96)	0.003, 0.004
Systolic blood pressure mmHg (mean, SD)	146 (4)	146 (3)	145 (4)	145 (4)	144 (4)	0.08, 0.09
Baseline HbgA1c (mean, SD)	10.66 (1.69)	10.75 (1.64)	11.06 (1.71)	10.40 (2.10)	10.28 (1.36)	0.206, 0.125
Final HbA1c (mean, SD)	8.71 (2.06)	10.60 (1.65)	8.38 (0.29)	7.49 (0.28)	6.25 (0.57)	0.001, 0.88
Diabetes duration, years (mean, SD)	22 (3.5)	20.3 (3.8)	21.4 (3.0)	22.9 (2.9)	23.7 (2.9)	0.12, 0.67
Total visits (mean, SD)	12 (3–21)	13 (3–22)	13 (3–22)	12 (4–21)	12 (4–21)	0.689, 0.685
Stroke (*N* [%])	1 (0.90)	1 (2.1)	0 (0)	0 (0)	0 (0)	1.0
Myocardial infraction (*N* [%])	5 (4.50)	3 (6.5)	0 (0)	1 (5.26)	1 (4.35)	0.700, 0.321
Cancer (*N* [%])	4 (3.60)	1 (2.1)	2 (8.7)	0 (0)	1 (4.3)	0.100, 0.685
Hypothyroidism (*N* [%])	14 (12.61)	4 (8.7)	4 (17.39)	3 (15.79)	3 (13.04)	0.081, 0.075
eGFR (mL/min/1.73 m^2^) mean	25.96 (11.1)	25.78 (10.7)	24.85 (9.2)	27.23 (14.7)	26.4 (10.9)	1.0, 95.0
CKD grade 3b (*N* [%])	39 (35.1)	16 (34.7)	8 (34.7)	5 (26.3)	10 (43.4)	0.001, 0.018
CKD grade 4 (*N* [%])	62 (54.9)	26 (56.5)	13 (56.5)	11 (57.63)	12 (52.1)	0.009, 0.008
CKD grade 5 (*N* [%])	10 (9.0)	4 (8.70)	2 (8.7)	3 (15.79)	1 (4.35)	0.090, 0.124
Diuretics (*N* [%])	12 (10.8)	4 (8.70)	5 (21.7)	1 (5.26)	2 (8.7)	0.038, 0.094
Acetylsalicylic acid (*N* [%])	17 (15)	10 (21)	2 (8.7)	2 (10.53)	3 (13.04)	0.071, 0.059
Hemoglobin, gr/L, mean (SD)	11.81 (1.89)	12.22 (1.56)	11.92 (1.86)	11.28 (2.64)	11.31 (1.71)	0.337, 0.088
Glucose, mg/dL, mean (SD)	189 (110)	204 (120)	185 (131)	176.42 (75.24)	174 (95)	0.9, 2.85
Urea, mg/dL, mean (SD)	105 (42.89)	109 (42.63)	94 (44)	108.95 (50.31)	106.37 (35.94)	0.464, 3.832
Creatinine, mg/dL, mean (SD)	2.70 (1.25)	2.70 (1.28)	2.77 (1.17)	2.66 (1.00)	2.66 (1.49)	0.022, 0.024
Sodium, mmol/L, mean (SD)	136 (3.70)	136 (3.58)	137 (3)	135 (3.7)	136 (3)	0.119, 0.310
Potassium mmol/L, mean (SD)	4.85 (0.66)	5.02 (0.54)	4.77 (0.57	4.50 (0.69)	4.90 (0.84)	0.064, 0.114
Chloride mg/dL, mean (SD)	103 (6)	102 (6)	104 (5.30)	103 (8.33)	103 (4)	0.188, 0.540
Phosphate, mg/dL, mean (SD)	4.21 (0.97)	4.16 (0.83)	3.91 (0.81)	4.28 (0.94)	4.66 (1.37)	0.162, 0.22
Calcium, mg/dL	9.05 (1.00)	9.23 (0.69)	9.15 (0.49)	8.81 (1.20)	8.75 (1.57)	0.178, 0.037
Serum albumin (gr/L)	3.83 (0.69)	3.83 (0.56)	3.83 (0.46)	3.79 (0.69)	3.86 (1.07)	0.005, 0.015
Total cholesterol (mg/dL)	168 (60)	157 (46)	163 (57)	182 (73)	182 (72)	0.9, 2.497
Triglycerides (mg/dL)	195 (105)	197 (95)	202 (91)	212 (151)	184 (74)	0.241, 0.67
Uric acid (mg/dL)	6.90 (2.19)	7.06 (2.15)	8.23 (2)	5.66 (1.81)	6.31 (1.4)	0.480, 0.499
Proteinuria on dipstick *n* (%) (defined as > 150)	30 (27.03)	9 (19.5)	6 (26.0)	8 (42.1)	7 (30.4)	0.043, 0.023
Proteinuria (grams, 24 h) mean (DE)	4.28 (2.92)	4.09 (3.76)	3.11 (2.01)	3.99 (2.31)	6.61 (1.86)	0.001, 0.27
Metabolic acidosis (*n*, %)	3 (2.70)	2 (4.3)	1 (4.35)	0 (0)	0 (0)	0.500, 0.288
Baseline Hgb (gr/dL) mean (SD)	11.81 (1.89)	12.22 (1.56)	11.92 (1.86)	11.28 (2.64)	11.31 (1.71)	0.337, 0.090
Number of HbA1c measurements, mean (SD)	4.92 (3.03)	4.70 (2.66)	4.74 (2.99)	5.68 (3.80)	4.91 (3.19)	0.159, 0.225
ACEi (*n*, %)	25 (22.2)	15 (32.6)	3 (133)	2 (10.5)	5 (21.74)	0.031, 0.034
ARB (*n*, %)	65 (58.5)	25 (54.3)	15 (65.3)	12 (63.1)	13 (56.5)	0.005, 0.007
MRA (*n*, %)	4 (3.6)	1 (2.1)	1 (4.35)	0 (0)	2 (8.7)	0.300, 0.793
B-blocker (*n*, %)	21 (18.9)	8 (17.3)	7 (30.4)	3 (15.7)	3 (13.0)	0.020, 0.039
Calcium antagonist (*n*, %)	37 (33.3)	14 (30.4)	6 (26.0)	6 (31.5)	11 (47.8)	0.007, 0.019
Statin (*n*, %)	93 (83.7)	37 (80.4)	20 (86.96)	16 (84)	20 (86.9)	0.003, 0.004
Bezafibrate (*n*, %)	7 (6.3)	4 (8.7)	0 (0)	1 (5.26)	2 (8.7)	0.178, 0.292
Allopurinol (*n*, %)	46 (41.4)	17 (36.9)	9 (39.13)	7 (36.3)	13 (56.52)	0.010, 0.014
Calcitriol (*n*, %)	16 (14.4)	6 (13.0)	2 (8.7)	1 (5.26)	7 (30.43)	0.001, 0.079
Calcium carbonate (*n*, %)	3 (2.7)	1 (2.1)	0 (0)	2 (10.53)	0 (0)	1.000, 0.577
Sodium bicarbonate (*n*, %)	7 (6.3)	3 (6.5)	2 (8.7)	1 (5.26)	1 (4.35)	0.035, 0.238
Erythropoietin (*n*, %)	11 (9.9)	4 (8.7)	2 (8.7)	4 (21.05)	1 (4.35)	0.001, 0.108
Insulin (*n*, %)	95 (85.5)	40 (86.9)	21 (91.3)	14 (73.68)	20 (86.96)	0.005, 0.004
SGLT2i (*n*, %)	22 (19.8)	12 (26.0)	5 (21.74)	3 (15.79)	2 (8.7)	0.032, 0.034
DPP4i (*n*, %)	7 (6.3)	2 (4.3)	1 (4.35)	3 (15.79)	1 (4.35)	0.142, 0.225
Metformin (*n*, %)	15 (13.5)	8 (17.3)	3 (13.0)	1 (5.26)	3 (13.04)	0.025, 0.075
Alpha blockers (*n*, %)	6 (5.4)	4 (8.7)	1 (4.3)	1 (5.26)	0 (0)	0.028, 0.223
Furosemide (*n*, %)	64 (57.6)	25 (54.3)	12 (52)	12 (63)	15 (65.22)	0.004, 0.008
Levothyroxine (*n*, %)	19 (17.1)	6 (13.0)	5 (21.7)	4 (21.0)	4 (17.39)	0.057, 0.047
Hypoglycemia events (*n*, %)	36 (32.4)	4 (3.6)	7 (6.3)	12 (10.8)	13 (11.7)	0.098, 0.026

*Note:* DPP4i, dipeptidyl peptidase type 4 inhibitors; Hgb, hemoglobin; HgbA1c, glycosylated hemoglobin; SGLT2i, sodium-glucose transporter inhibitors.

Abbreviations: ACEi, angiotensin-converting enzyme inhibitors; ARB, antagonist receptor blockers; CKD, chronic kidney disease; MRA, mineralocorticoid receptor antagonist.

**Table 2:** Odds ratio of primary and secondary objectives according to HbA1c groups at the end of the follow-up.

	HbA1c > 75 mmol/mol (≥ 9.0%)	HbA1c 74–64 mmol/mol (8.9%–8.0%)	HbA1c 64–53 mmol/mol (7.9%–7.0%)	HbA1c < 52 mmol/mol (< 7.0%)
		OR (95% CI) *p* value	OR (95% CI) *p* value	OR (95% CI) *p* value
*Primary objective*				
MAKE25^∗^	(Ref)	1.47 (0.51–4.27) 0.48	1.80 (0.55–5.86) 0.32	1.47 (0.51–4.27) 0.48
MAKE40^∗∗^	(Ref)	1.19 (0.31–4.59) 0.73	1.02 (0.28–3.71) 0.97	1.43 (0.39–5.23) 0.49

*Secondary objectives*				
Death	(Ref)	5.35 (0.63–45.13) 0.12	1.30 (0.31–5.43) 0.72	1.62 (0.39–6.68) 0.50
New requirement of KRT	(Ref)	1.11 (0.75–2.43) 0.85	0.78 (0.75–2.43) 0.54	1.11 (0.75–2.43) 0.85
Worsening of kidney function denoted by ≥ 25% decline in the eGFR from baseline	(Ref)	1.31 (0.47–3.62) 0.60	1.44 (0.48–4.31) 0.51	1.31 (0.47–3.62) 0.60
Worsening of kidney function denoted by ≥ 40% decline in the eGFR from baseline	(Ref)	1.00 (0.82–3.34) 1.00	1.05 (0.82–3.34) 0.92	2.10 (0.82–3.34) 0.16

Abbreviations: eGFR, estimated glomerular filtration rate; KRT, kidney replacement therapy; MAKE, major adverse kidney event.

^∗^Death, KRT requirement or ≥ 25% eGFR decline from baseline.

^∗∗^Death, KRT requirement or ≥ 40% eGFR decline from baseline.

**Table 3:** Percentage change in HbA1c, taking as a reference the −1.5%–0% group, and the risk of KRT.

HbA1c change (%)	No. with event (KRT start)	*n*	OR (95% CI)	*p* value
< −2.5	11	38	2.24 (0.63–8.02)	0.21
−2.5–−1.5	8	25	2.59 (0.67–10.05)	0.17
−1.5–0	4	26	1.00 (reference)	NA
> 0	8	22	3.14 (0.79–12.43)	0.10

## Data Availability

The files and data are in the physical and electronic archives of the Civil Hospital of Guadalajara Fray Antonio Alcalde and can be requested with prior authorization. All data generated or analyzed during this study are included in this article. Further inquiries can be directed to the corresponding author.

## Supporting Information

Supporting Figure 1. Total cohort eGFR trajectory.

Supporting Figure 2. Alluvial plot of the frequency of MAKE according to the different HbA1c groups at the end of the follow-up.

Supporting Figure 3. Mortality analyzed as the accumulated survival during the 60 months of follow-up among the HbA1c groups.

Supporting Table 1. MAKE frequency and its individual components according to final HbA1c.
