# Supplementary material for: Impact of HbA1c Reduction on Major Kidney Outcomes in Type 2 Diabetes With Poor Glycemic Control and Advanced CKD
Source: Int J Endocrinol. 2025 May 4;2025:9919963. doi: 10.1155/ije/9919963 (PMC12066180; doi:10.1155/ije/9919963)
Supplement: Supporting Information — Additional supporting information can be found online in the Supporting Information section. [file 9919963.f1.docx]

**Supp Figure 1.** Total cohort eGFR trajectory and according to HbA1c groups.


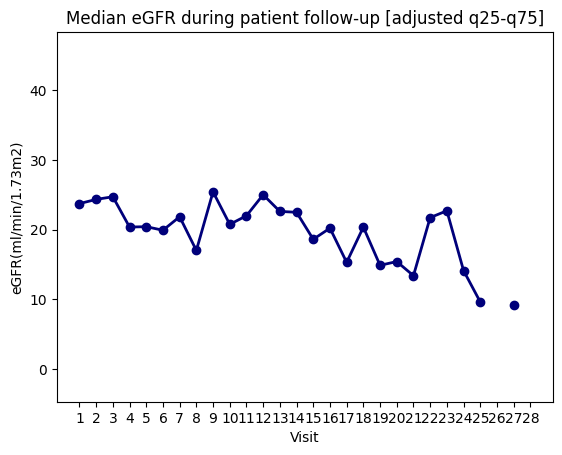


**Supplemental Figure 2.** Alluvial plot of the frequency of MAKE according to the different HbA1c groups at the end of the follow-up.


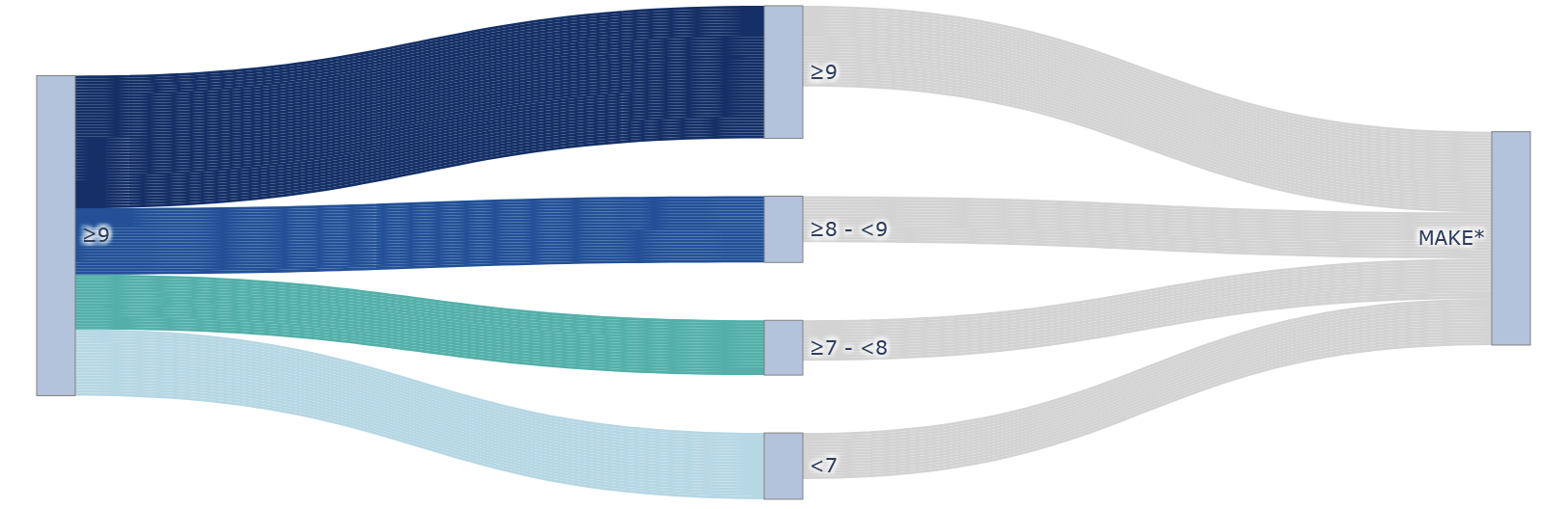


**Supplemental Figure 3.** Mortality analyzed as the accumulated survival during the 60 months of follow-up among the HbA1c groups.


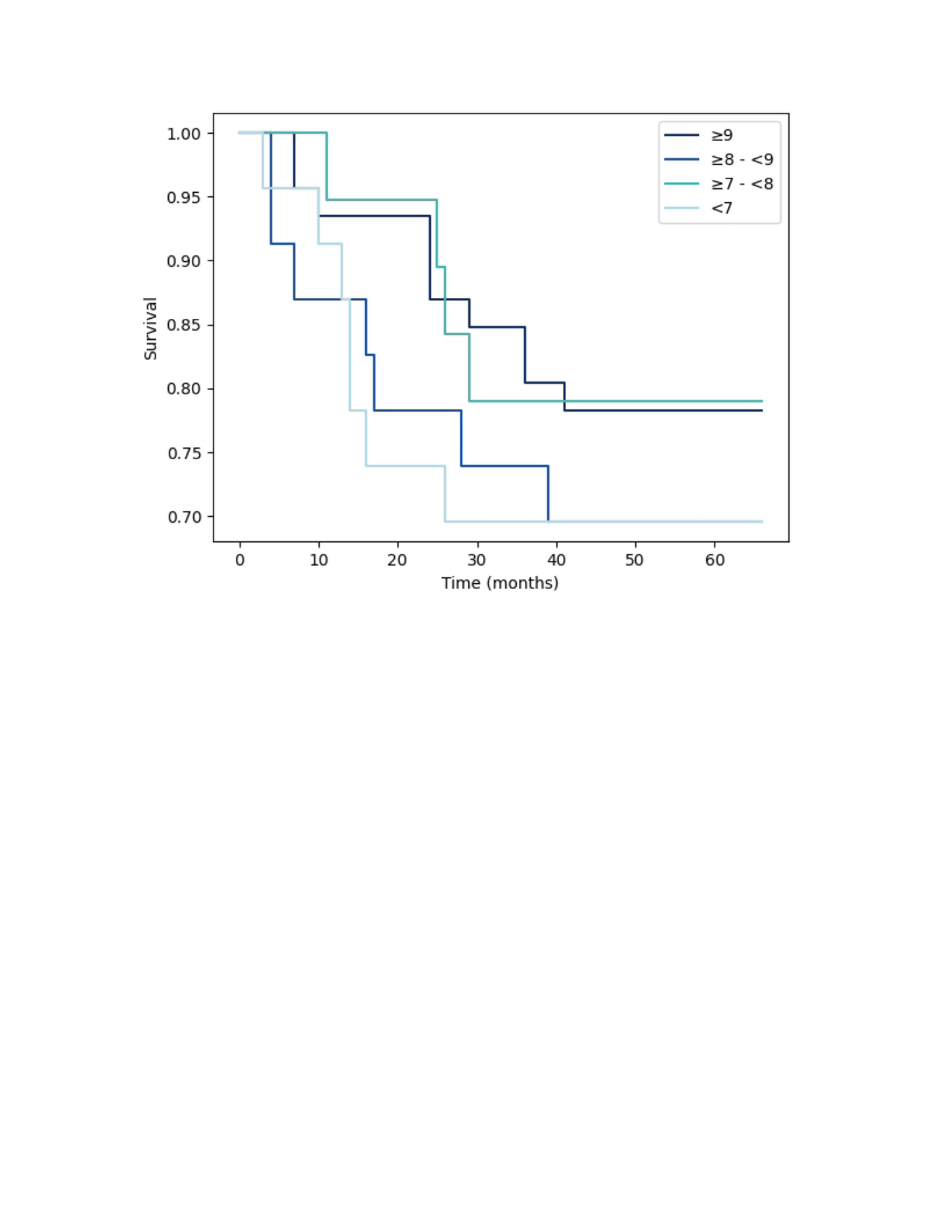


**Suppl tab 1.** MAKE frequency and its individual components according to final HbA1c.

| HbA1c group | MAKE25 | MAKE40 | Start KRT | Death | 25% eGFR decline | 40% eGFR decline |
| --- | --- | --- | --- | --- | --- | --- |
| >75 mmol/mol, >9% | 28 | 24 | 13 | 9 | 25 | 14 |
| 74-64 mmol/mol, 8 to 9% | 16 | 13 | 7 | 1 | 14 | 7 |
| 64-53 mmol/mol , 7 to 8% | 14 | 10 | 4 | 3 | 12 | 6 |
| <52 mmol/mol, <7% | 16 | 14 | 7 | 3 | 14 | 11 |
| Total | 74 | 61 | 31 | 16 | 65 | 38 |

eGFR, estimated glomerular filtration rate; KRT, kidney replacement therapy; MAKE, major adverse kidney events.
